# Atorvastatin and Nitrofurantoin Repurposed in the Context of Breast Cancer and Neuroblastoma Cells

**DOI:** 10.3390/biomedicines11030903

**Published:** 2023-03-15

**Authors:** Catarina Moura, Ana Salomé Correia, Mariana Pereira, Eduarda Ribeiro, Joana Santos, Nuno Vale

**Affiliations:** 1 OncoPharma Research Group, Center for Health Technology and Services Research (CINTESIS), Rua Doutor Plácido da Costa, 4200-450 Porto, Portugal; 2 CINTESIS@RISE, Faculty of Medicine, University of Porto, Alameda Professor Hernâni Monteiro, 4200-319 Porto, Portugal; 3 ICBAS-School of Medicine and Biomedical Sciences, University of Porto, Rua Jorge Viterbo Ferreira, 228, 4050-313 Porto, Portugal; 4 Department of Community Medicine, Information and Health Decision Sciences (MEDCIDS), Faculty of Medicine, University of Porto, Rua Doutor Plácido da Costa, 4200-450 Porto, Portugal

**Keywords:** doxorubicin, drug combination, drug repurposing, MCF-7 cells, SH-SY5Y cells, atorvastatin, nitrofurantoin

## Abstract

Chemotherapy still plays a central role in the treatment of cancer. However, it is often accompanied by off-target effects that result in severe side-effects and development of drug resistance. The aim of this work was to study the efficacy of different repurposed drugs on the viability of MCF-7 and SH-SY5Y breast cancer and neuroblastoma cells, respectively. In addition, combinations of these repurposed drugs with a classical chemotherapeutic drug (doxorubicin) were also carried out. The cytotoxic effects of the repurposed drugs were evaluated individually and in combination in both cancer cell lines, assessed by MTT assays and morphological evaluation of the cells. The results demonstrated that atorvastatin reduced the viability of both cell lines. However, nitrofurantoin was able to induce cytotoxic effects in MCF-7 cells, but not in SH-SY5Y cells. The combinations of the repurposed drugs with doxorubicin induced a higher inhibition on cell viability than the repurposed drugs individually. The combination of the two repurposed drugs demonstrated that they potentiate each other. Synergism studies revealed that the combination of doxorubicin with the two repurposed drugs was more effective in SH-SY5Y cells, compared to MCF-7 cells. Taken together, our preliminary study highlights the potential use of atorvastatin and nitrofurantoin in the context of breast cancer and neuroblastoma.

## 1. Introduction

Cancer is a disease that involves the abnormal and uncontrolled growth of cells. The fundamental approach of any cancer therapy is to suppress tumor growth, control metastases, and prevent relapse after elimination, thereby prolonging the patient’s life. Conventionally used methods of cancer therapy include surgery, chemotherapy, and radiation therapy. Each method has its limitations and, therefore, is often not sufficient to produce satisfactory therapeutic results in patients, which leads to new studies being conducted to try to find new forms of treatments [1].

According to the World Health Organization (WHO), breast cancer is one of the main cancers affecting individuals worldwide, with 2.26 million new cases diagnosed in 2020 [2], which corresponds to the second cause of death from cancer in women [3]. It is assumed that one in eight women in the world will develop mammary gland cancer, and that only 5–10% of all cases of this cancer are caused by genetic diseases, while the remaining 90–95% of cases are linked to environmental and lifestyle factors [4].

Although treatment with single compounds can be beneficial, several recent studies have reported better results in combinations of two or more compounds compared to using a single compound. The combination of drugs has been used in several areas, one of them being cancer. When combining two or more drugs, the main goal is to achieve positive interaction effects that show superior evidence of the beneficial combination of two or more drugs compared to each drug individually, i.e., to achieve more with less [5]. The effects of the combination can be synergistic, antagonistic, or potentiating [6].

Several regimens that include two or more molecularly targeted agents have already been approved, and a number of combinations are in late-stage clinical development. The first combination of two HER2 (also known as ERBB2)-targeted drugs pertuzumab and trastuzumab, along with the chemotherapy agent docetaxel, was approved by the FDA in June 2012 for metastatic breast cancer. The second FDA-approved combination was the combination of a BRAF inhibitor and a MAPK/ERK kinase inhibitor (MEK), which was granted an accelerated approval by the FDA in January 2014 for the treatment of unconventional or metastatic BRAFV600E/K melanoma; both agents were developed by GlaxoSmithKline (GSK) and acquired by Novartis in March 2015. In October 2015, the FDA granted accelerated approval to the first combination immune checkpoint inhibitor, the programmed cell death protein 1 (PD1) inhibitor nivolumab and the cytotoxic T lymphocyte antigen 4 (CTLA4) inhibitor ipilimumab, for BRAFV600 unresectable or metastatic wildtype melanoma [7].

Drug repurposing refers to the application of a drug for another indication than was originally approved and has received increasing interest as an alternative strategy to the synthesis of new drugs. A major advantage of this use is that extensive data are often available, which reduces the need for additional studies to investigate the pharmacokinetic properties and toxicity of drugs. The repurposing of drugs for a new indication may, however, be accompanied by side-effects not previously found, which will require the validation of a new clinical trial [8].

The combination of a reference drug has the objective of already having a safe starting point, since the reference drug already has antitumor activity that is guaranteed in tumor cells. The combination with the repurposed drug, which already has an acceptable toxicological profile, aims to improve the activity of the reference drug and simultaneously reduce its therapeutic dose [9].

In this work, we aimed to focus on drug repurposing and drug combination studies, using atorvastatin (a statin), nitrofurantoin, and doxorubicin (DOX). We aimed to develop a combination model in which both repurposed drugs have synergistic effects when combined with a clinically used chemotherapeutic drug. We decided to choose atorvastatin since it has shown promising results in prostate cancer; moreover, in one study, it inhibited prostate cancer cell growth in a concentration-dependent manner [10]. Nitrofurantoin was chosen because it is a synthetic antibiotic which has been shown to have potential toxic effects attributable to the nitro group (NO_2_) attached to the furan ring. The nitro group gives this molecule a toxicophore function, which acts as an electron acceptor, thereby inhibiting enzymes involved in pyruvate metabolism, an essential pathway of cellular metabolism. Nitrofurantoin has also been shown to be cytotoxic against cancer cells, inhibiting proliferation of human leukemia, colon, cervix, and prostate cancer cell lines [11].

There are few references to the interaction between the drugs nitrofurantoin together with atorvastatin, but a possible indication is that it may increase the risk of nerve damage. We intended to understand the effect of these drugs on cancer cells alone and then combined with a potent reference drug doxorubicin, as well as a combination of the three. No work of this kind has ever been performed, and new evidence was found to better understand the combination of nitrofurantoin with atorvastatin.

**Figure 1:**
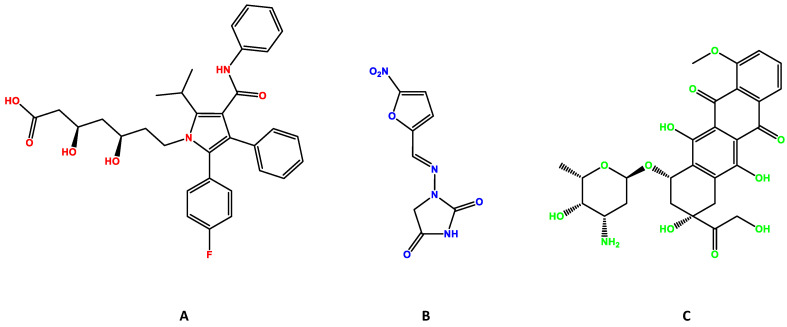
Chemical structure of the drugs applied in this project: (**A**) atorvastatin, (**B**) nitrofurantoin, and (**C**) doxorubicin.

Statins belong to a group of drugs that work by decreasing blood cholesterol levels through specific inhibition of the enzyme 3-hydroxy-3-methylglutaryl coenzyme A (HMG-CoA) reductase. In addition to these effects on lipid metabolism, statins induce immunomodulatory, anti-inflammatory, and antioxidant activity. During the last few years, antineoplastic effects of statins have also been reported [12]. Atorvastatin (Figure 1A) is one of the most frequently prescribed statins for the prevention of cardiovascular and cerebrovascular diseases. This drug also shows antiproliferative effects on different cancer cells, including breast cancer cells. Thus, atorvastatin has gained increasing interest as a potential therapeutic agent for use as an anticancer treatment. Although the exact mechanism of its antiproliferative effects is currently unknown, atorvastatin both modifies the cell cycle and induces growth suppression or apoptosis of malignant cells. Furthermore, the lipophilic nature of atorvastatin allows it to easily cross the cell membrane and induce these effects [12]. In one study, atorvastatin was shown to have proapoptotic and antimetastatic effects on prostate cancer cells. Parikh et al. hypothesized that atorvastatin may induce autophagy-associated cell death in PC3 cells. However, the biological mechanisms underlying the anticancer effects of atorvastatin have yet to be elucidated [10].

Nitrofurantoin (Figure 1B), an antibiotic drug [13], is a synthetic nitrofuran derivative of hydantoin used for the prevention and treatment of urinary tract infections. The mode of action of this drug involves the reduction of the nitro group by bacterial flavoenzymes producing reactive intermediates and the formation of hydroxyl radicals. These radicals can interact with DNA, resulting in inhibition of nucleic acid synthesis and breaks of single- and double-stranded DNA. Nitrofurantoin has been shown to be cytotoxic against cancer cells, inhibiting proliferation of human leukemia, colon, cervical, and prostate cancer cell lines [11].

Doxorubicin (DOX) (Figure 1C) is an anthracycline antibiotic, isolated from the species *Streptomyces peucetius*, and it is used effectively in several types of cancer [14]. In the cancer cell, DOX intercalates into the DNA and disrupts topoisomerase-II mediated DNA repair. This also generates free radicals that damage cell membranes, DNA, and proteins [1]. Unfortunately, despite being highly effective, doxorubicin is also not selective for cancer cells, meaning its use is significantly limited due to its toxicity [14]. Although DOX is a popular anticancer drug, its clinical results are still unsatisfactory due to the dominant effect of drug resistance mechanisms. In this way, if a higher dosage is prescribed to increase its effectiveness, it may have adverse side-effects on normal tissue cells, primarily affecting the heart and kidneys [1].

As mentioned earlier, doxorubicin is a widely used drug in the treatment of various cancers. Thus, we decided to choose two different cancer cell lines for this work, MCF-7 and SH-SY5Y. MCF-7 cells and SH-SY5Y cells are, respectively, human breast cancer and neuroblastoma cells. Both cell lines are epithelial and were collected from metastatic tumors, having high proliferative capabilities [15,16]. These cell lines represent commonly used human cell lines in research, particularly for the study of breast cancer and neurological diseases, such as Parkinson’s disease [17]. Indeed, the MCF-7 cell line is the most studied human breast cancer cell line in the world [18]. In fact, drug repurposing studies are frequently performed in these two cell lines [19,20].

**Scheme 1:**
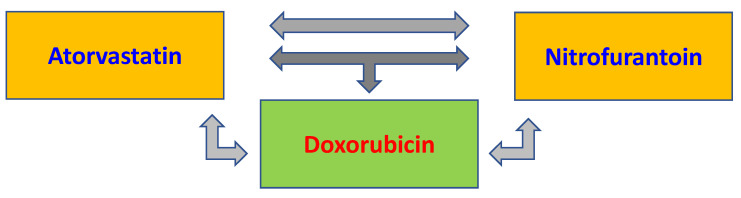
Drug combination model used in this project.

Thus, the main goal of this work was to evaluate the efficacy of atorvastatin and nitrofurantoin on the viability of MCF-7 and SH-SY5Y cells (Scheme 1). We also aimed to analyze the combination of doxorubicin (a reference drug already used in the treatment of breast cancer) with the mentioned repurposed drugs and evaluate whether together these drugs had a greater inhibition in the breast cancer line MCF-7 or in human neuroblastoma SH-SY5Y, and consequently compare the drug combination with the drugs individually.

## 2. Materials and Methods

### 2.1. Drug Solutions

For the treatment of the cells with the drugs under study, DOX (Cayman Chemical Company cat. 15007, Cayman Europe, Tallinn, Estonia), ATOR (Sigma-Aldrich cat. PHR1422-1G, Sintra, Portugal), and NITRO (Cayman Chemical Company cat. 23510, Cayman Europe, Tallinn, Estonia), were dissolved in dimethyl sulfoxide (DMSO). A stock solution of each compound was prepared at a concentration of 100 mM for ATOR, at a concentration of 10 mM for DOX, and at a concentration of 200 mM for NITRO. In addition to these stock concentrations, a new stock solution for 200 mM ATOR was then needed. All these stock solutions were kept in the refrigerator at approximately 4 °C. The concentrations used in each assay for DOX were 0.01, 0.1, 1, 5, and 10 μM; those for ATOR and NITRO were 0.1, 1, 10, 25, 50, and 100 μM.

### 2.2. Cell Culture

The experimental work was performed with MCF-7 and SH-SY5Y (ATCC, American Type Culture Collection, Manassas, VA, USA) cell lines. The cells were incubated at 37 °C in a humidified atmosphere with 95% air and 5% CO_2_. Cells were cultured Dulbecco’s modified Eagle medium (DMEM), supplemented with 10% fetal bovine serum (FBS) and 1% penicillin/streptomycin mixture (1000 U/mL; 10 mg/mL). For maintenance, cells were cultured in a monolayer and sub-cultured by trypsinization in the same medium when a confluence of ~80% was reached. Cells were maintained in logarithmic growth phase at all timepoints.

### 2.3. MTT Reduction Assay

Cells were plated in 96-well plates at a seeding density of 5.0 × 10^4^ cells/mL, kept in a 37 °C incubator for 24 h before exposure to the drug. After this time, the cell culture media were replaced with 200 μL of media containing drugs with different treatments and different concentrations for 48 h. The cells were kept at 37 °C for the mentioned time. Then, the cell medium was removed, and 100 μL of MTT solution (0.5 mg/mL in PBS) was added to each well. Subsequently, the cells were incubated at 37 °C for 2 h, protected from light. At the end of this time, MTT was removed, and 100 μL of DMSO was added to each well. The last step consisted of absorbance readings at 570 nm in an automated microplate reader (Sinergy HT, BioTek Instruments, Winooski, VT, USA) to evaluate the effects with the drugs alone and in combination on the cell viability of MCF-7 and SH-SY5Y cells.

### 2.4. Evaluation of the Effect of Drugs

Half of the maximum inhibitory concentration (IC_50_) value was first determined for each drug alone in MCF-7 and SH-SY5Y cells. The concentrations of the drugs used ranged from 0.1 to 100 µM for single drug treatment. The combination studies were performed by combining DOX (Drug 1) with the repurposed drugs (Drug 2), combining DOX with two repurposed drugs, and combining the two repurposed drugs with each other. Only the drugs that showed the most promising pharmacological profile, such as ATOR and NITRO, were tested in combination with DOX and presented in this paper. The concentrations of both Drug 1 and Drug 2 were variable.

### 2.5. Cell Morphology Visualization

After the treatment with the drugs, the morphological characteristics of MCF-7 and SH-SY5Y cells were captured using a Leica DMI 6000B microscope coupled to a Leica DFC350 FX camera (Leica Microsystems, Wetzlar, Germany). The plate containing the cells was placed on the microscope, and the images of the cells were analyzed on the computer using Leica Las X imaging software (v3.7.4) (Leica Microsystems, Wetzlar, Germany).

### 2.6. Data Analysis

GraphPad Prism 8 (GraphPad Software Inc., San Diego, CA, USA) was used to create bar graphs of cell viability and to produce concentration–response curves by nonlinear regression analysis. The viability of cells treated with each drug was normalized to the viability of control cells and cell viability fractions were plotted versus drug concentrations on a logarithmic scale.

### 2.7. Statistical Analysis

Statistical analysis was performed in all experiments. The results are expressed as the arithmetic mean ± standard error of the mean (SEM) for n experiments performed, explicit in the legends of the graphs. Differences between the treated cells and the corresponding untreated control were tested using one-way ANOVA.

### 2.8. Synergism Studies

Using the CompuSyn software (version 1.0; ComboSyn, Paramus, NJ, USA) and through the Chou–Talalay equation, the combination index (CI) and the fractional effect (Fa) of the combinations were assessed, using a non-fixed ratio. In this context, a CI inferior to 1 indicates synergism between the drugs, while values equal to 1 indicate additivity, and CI values superior to 1 indicate antagonism. The Fa ranges between 0 and 1, representing cellular death, with 0 being no cell death and 1 being total cell death.

## 3. Results and Discussion

### 3.1. Effect of the Repurposed Drugs on MCF-7 and SH-SY5Y Cell Viability

**Figure 2:**
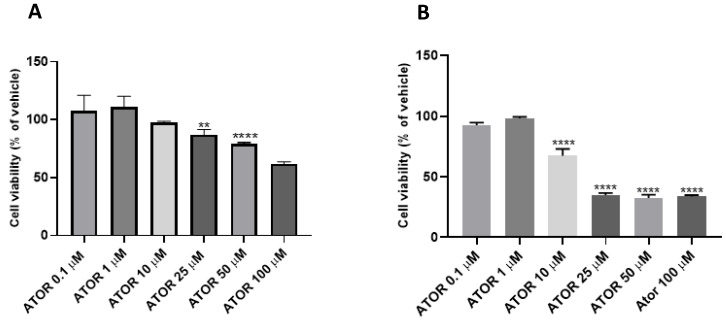
Effect of ATOR on the viability of MCF-7 (**A**) and SH-SY5Y (**B**) cells. The cells were cultured in the presence of increasing concentrations of ATOR. After 48 h, MTT assay was performed to measure cell viability. Values are expressed as percentages and represent the means ± SEM. Each experiment was performed three times independently (n = 3). One-way ANOVA was used as statistical test. Statistically significant ** *p* < 0.01, and **** *p* < 0.0001 vs. vehicle.

To evaluate the effects of atorvastatin (ATOR) on the viability of MCF-7 and SH-SY5Y cells, the cells were treated with this drug in a concentration range between 0.1 and 100 µM for 48 h. The percentage cell viability was evaluated by MTT assay (Figure 2).

**Figure 3:**
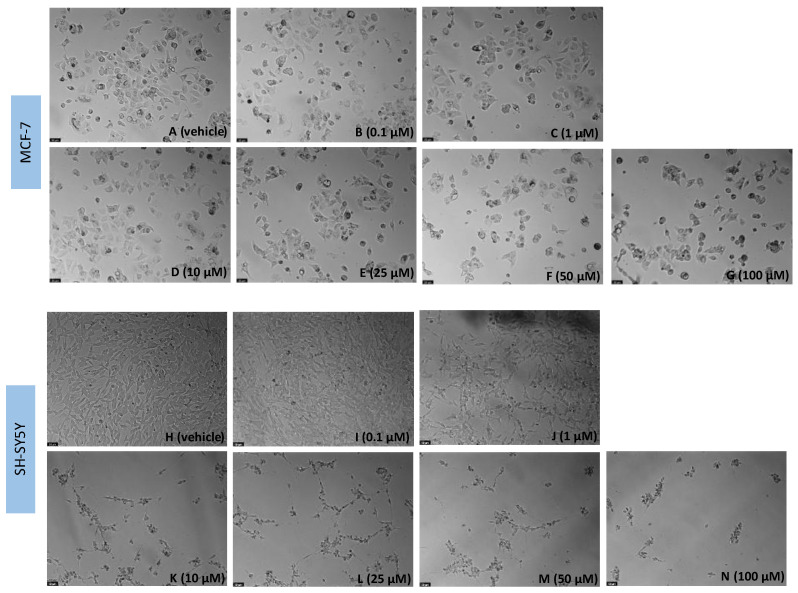
Microscopic visualization of the effects of ATOR on the morphology of MCF-7 and SH-SY5Y cells over 48 h. Cells were treated with (**A**,**H**) 0.1% DMSO (vehicle) or (**B**,**I**) 0.1 µM, (**C**,**J**) 1 µM, (**D**,**K**) 10 µM, (**E**,**L**) 25 µM, (**F**,**M**) 50 µM, or (**G**,**N**) 100 µM ATOR. Scale bar: 50 µm; 100× total magnification.

Our results demonstrate that ATOR had a significant inhibitory effect for the highest concentrations of 25, 50, and 100 µM (Figure 2 and Figure 3E–G,L–N) for both cells tested; for SH-SY5Y cells, the effect was much more accentuated, which evidences that ATOR had greater cytotoxic effects in these cells, compared to MCF-7 cells. Being neuronal cells, SH-SY5Y cells may be more sensitive to the effects of this drug, explaining these differences between cell lines. Indeed, in a study, statins demonstrated to induce apoptosis in SH-SY5Y cells by reducing the levels of dolichol, required for the biosynthesis of biologically important N-linked glycoproteins [21].

For SH-SY5Y cells, viability values of about 35%, 33%, and 34% were obtained for the 25, 50, and 100 µM concentrations, respectively, while, for MCF-7 cells, the cell viability values obtained were 87%, 79%, and 62%, respectively, for the 25, 50, and 100 µM concentrations of ATOR. These cell viability values were also confirmed by cell morphology (Figure 3), whereby, at these concentrations, the cells were rounded and smaller in shape compared to the control (Figure 3A), which shows that these cells are unviable and that, consequently, ATOR had a concentration-dependent inhibitory effect on MCF-7 and SH-SY5Y cells, with this anticancer effect being highest for SH-SY5Y cells. Therefore, it was possible to obtain an IC50 for ATOR for both cell lines tested; with MCF-7, the IC50 obtained was 37.95 µM, whereas, for SH-SY5Y, an IC50 of 10.10 µM was obtained, as evidenced in Table 1. These findings demonstrated that ATOR is a repurposed drug intended for the reduction in blood cholesterol, but it evidenced anticancer effects in MCF-7 and SH-SY5Y cells. Indeed, studies indicate that the growth/survival of some types of cancer depend on the mevalonate pathway, being vulnerable to statin therapy because these drugs inhibit HMG-CoA reductase, an important enzyme of the mevalonate pathway. In fact, statins have been shown to induce tumor-specific apoptosis, being also associated with reduced cancer risk [22].

**Figure 4:**
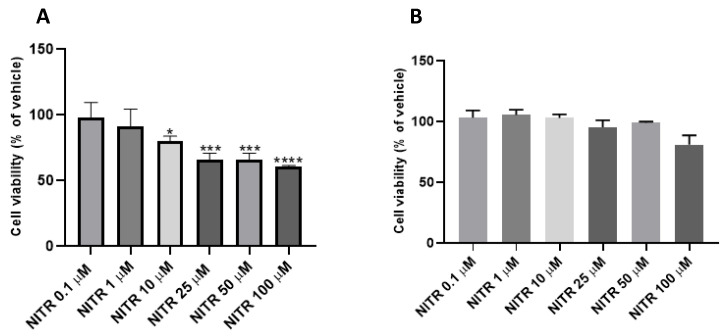
Effects of NITR on cell viability of MCF-7 (**A**) and SH-SY5Y (**B**) cells. The cells were cultured in the presence of increasing concentrations of NITR. After 48 h, MTT assay was performed to evaluate cell viability. Values are expressed as percentages and represent means ± SEM. Each experiment was performed three times independently (n = 3). One-way ANOVA was used as statistical test. Statistically significant * *p* < 0.05, *** *p* < 0.001, and **** *p* < 0.0001 vs. vehicle.

The effects of nitrofurantoin (NITR) were evaluated on the viability of MCF-7 and SH-SY5Y cells; for this purpose, cells were treated with NITR in a concentration range between 0.1 and 100 µM for 48 h. The percentage cell viability was assessed by MTT assay (Figure 4).

**Figure 5:**
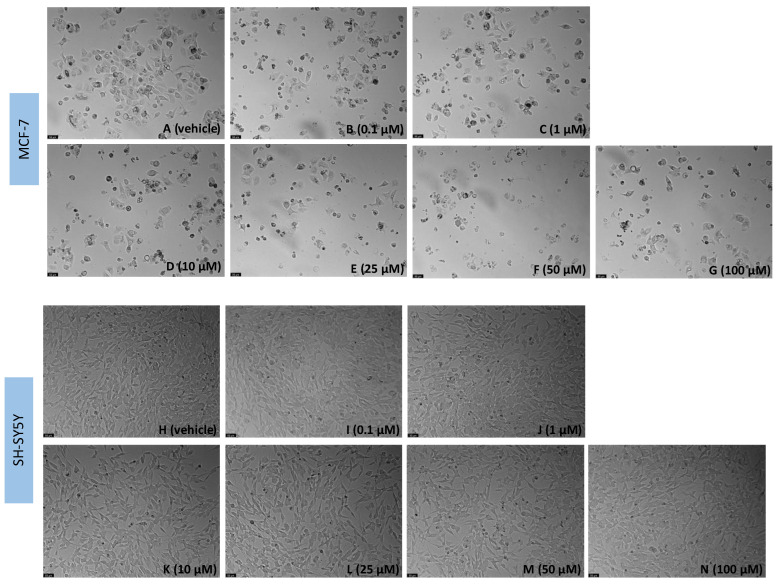
Microscopic visualization of the effects of NITR on the morphology of MCF-7 and SH-SY5Y cells over 48 h. Cells were treated with (**A**,**H**) 0.1% DMSO (vehicle) or (**B**,**I**) 0.1 µM, (**C**,**J**) 1 µM, (**D**,**K**) 10 µM, (**E**,**L**) 25 µM, (**F**,**M**) 50 µM, or (**G**,**N**) 100 µM NITR. Scale bar: 50 µm; 100× total magnification.

The morphology of MCF-7 and SH-SY5Y cells treated with different concentrations of NITR for 48 h is evidenced in Figure 5.

Our results demonstrate that NITR was effective in reducing the cell viability of MCF-7 cells (Figure 4A) for almost all concentrations (10, 25, 50, and 100 µM), for which viability percentages of 80%, 66%, 66%, and 61%, respectively, were obtained. In Figure 5, this effect can also be observed, revealing that the morphology of MCF-7 cells for the previously mentioned concentrations of the NITR was different from the morphology of the control cells (Figure 5A); that is, in the images, it can be observed that there are fewer cells compared to the control and that the cells have a rounded and smaller shape, a characteristic of cells that are unviable. For the SH-SY5Y cell line, a very effective inhibitory effect was not observed, since there was no noticeable decrease in cell viability for any of the concentrations tested. The only concentration that showed a decrease in cell viability was 100 µM, but it only reached a percentage viability of about 81%, and the remaining concentrations tested were close to 100% cell viability. Thus, for MCF-7 cells, it was possible to obtain an IC50 of 5.7 µM (Table 1), a very low and very good value, since this drug is a repurposed drug used for the prevention and treatment of urinary tract infections, now demonstrating anticancer effects for these cells. For the SH-SY5Y cell line, it was not possible to obtain an IC50, since the results showed that NITR in these cells did not have an inhibitory effect on cell viability. Indeed, this pronounced effect on MCF-7 cells may be explained by the evidence that nitrofurantoin interacts with the human BCRP (breast cancer resistance protein) (https://pubmed.ncbi.nlm.nih.gov/15709111/, accessed on 1 September 2022). However, there are few studies about the effect of this drug in both breast cancer and neuroblastoma, making it interesting to explore the differential effects of this drug in this cell cultures. Nevertheless, some studies demonstrated cytotoxic activity of this drug. For example, in HL-60 leukemia cells, this drug upregulated BAX and downregulated BCL-xL expression, inducing apoptosis [11].

### 3.2. Effect of Different Combinations of DOX and Repurposed Drugs on the Cell Viability of MCF-7 and SH-SY5Y Cells

**Figure 6:**
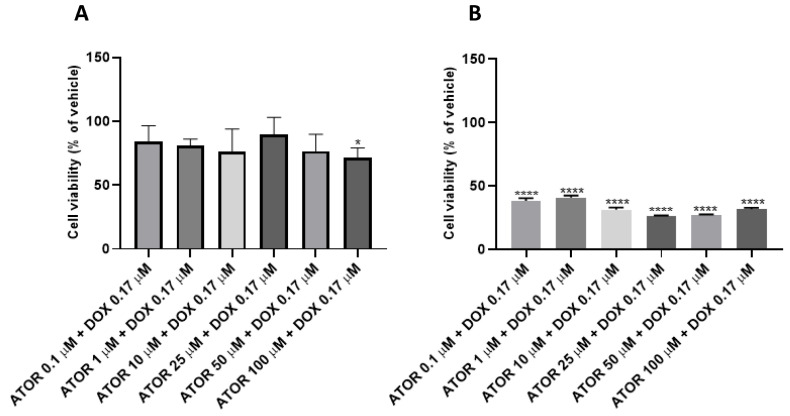
Effects of combining DOX with ATOR on cell viability of MCF-7 (**A**) and SH-SY5Y (**B**) cells. Cells were cultured in the presence of a single concentration of DOX (0.17 µM) and with increasing concentrations of ATOR. After 48 h, the MTT assay was performed to measure cell viability. Values are expressed as percentages and represent means ± SEM. Each experiment was performed three times independently (n = 3). One-way ANOVA was used as statistical test. Statistically significant * *p* < 0.05, and **** *p* < 0.0001 vs. vehicle.

To evaluate the different combinations of DOX with ATOR on the viability of MCF-7 and SH-SY5Y cells, cells were treated with 0.17 µM DOX (IC50 obtained for doxorubicin by the research group) [23] and with ATOR in a concentration range between 0.1 and 100 µM for 48 h. The percentage cell viability was assessed by MTT assay (Figure 6).

**Figure 7:**
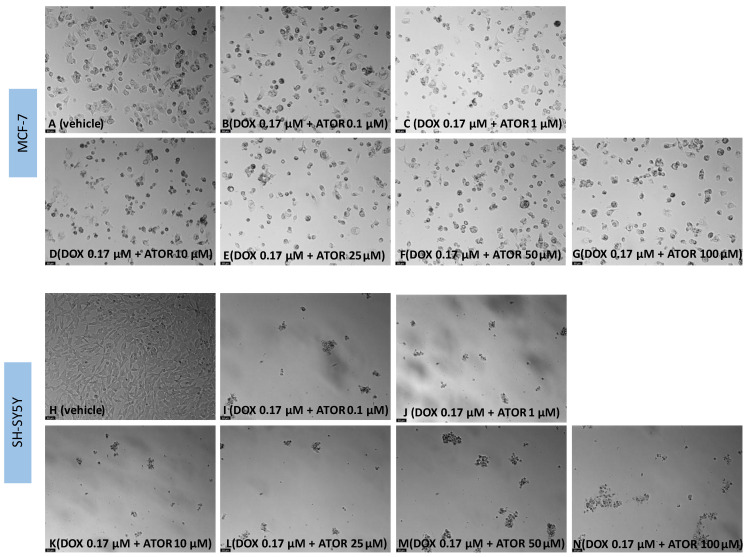
Microscopic visualization of the effects of combining DOX with ATOR on the morphology of MCF-7 and SH-SY5Y cells for 48 h. Cells were treated with (**A**,**H**) 0.1% DMSO (control), (**B**,**I**) 0.17 µM DOX + 0.1 µM ATOR, (**C**,**J**) 0.17 µM DOX + 1 µM ATOR, (**D**,**K**) 0, 17 µM DOX + 10 µM ATOR, (**E**,**L**) 0.17 µM DOX + 25 µM ATOR, (**F**,**M**) 0.17 µM DOX + 50 µM ATOR, or (**G**,**N**) 0.17 µM DOX + 100 µM ATOR. Scale bar: 50 µm; 100× total magnification.

Through the results obtained for the combination of DOX with ATOR for the SH-SY5Y cell line (Figure 6B and Figure 7), it is possible to observe that this combination was very beneficial for both DOX and ATOR, since, for almost all the results obtained (except ATOR 100 µM + DOX 0.17 µM), the cell viability decreased greatly compared to ATOR individually, and the cell viability for all combinations always remained below 50%. A possible explanation for these achievements may be that DOX may increase the sensitivity of cells to the effect of other drugs, potentiating their apoptotic effects. Indeed, chemosensitization is a strategy to overcome chemoresistance, based on the use of one drug to potentiate the activity of another [24].

The combination for this cell line that obtained the best results was 0.17 µM DOX with 25 µM ATOR, which achieved a cell viability of about 26%, i.e., a cell death rate of about 74%. Contrary to SH-SY5Y cells, MCF-7 cell viability did not stay below 50% for any of the tested combinations, but this combination still managed to be very beneficial for ATOR, since, for almost all combinations, it was possible to decrease cell viability and consequently increase cell death, except for the concentration of 0.17 µM DOX with 25 µM ATOR, where this decrease was not visible and, therefore, cell viability remained the same for the combination and for ATOR alone. Thus, we can see that these two drugs together showed quite marked cytotoxic effects in SH-SY5Y cells and little effect in MCF-7 cells compared to the drugs tested individually; consequently, each drug was able to potentiate the other to have better effects, increasing cell death in the cells tested.

**Figure 8:**
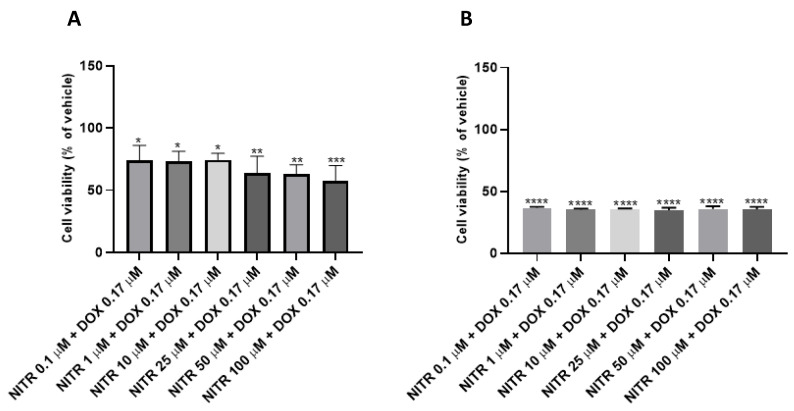
Effects of combining DOX with NITR on cell viability of MCF-7 (**A**) and SH-SY5Y (**B**) cells. The cells were cultured in the presence of a single concentration of DOX (0.17 µM) and with increasing concentrations of NITR. After 48 h, MTT assay was performed to measure cell viability. Values are expressed as percentages and represent means ± SEM. Each experiment was performed three times independently (n = 3). One-way ANOVA was used as statistical test. Statistically significant * *p* < 0.05, ** *p* < 0.01, *** *p* < 0.001, and **** *p* < 0.0001 vs. vehicle.

The effects of different combinations of DOX with NITR were evaluated on the viability of MCF-7 cells; for this purpose, MCF-7 cells were treated with 0.17 µM DOX and with NITR in a range of concentrations between 0.1 and 100 µM for 48 h. The percentage cell viability was assessed by MTT assay (Figure 8).

**Figure 9:**
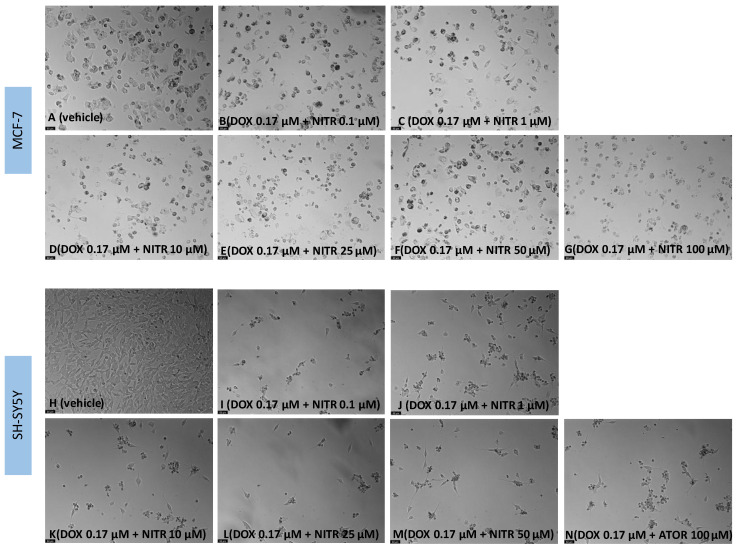
Microscopic visualization of the effects of combining DOX with NITR on the morphology of MCF-7 cells for 48 h. Cells were treated with (**A**,**H**) 0.1% DMSO (control), (**B**,**I**) 0.17 µM DOX + 0.1 µM NITR, (**C**,**J**) 0.17 µM DOX + 1 µM NITR, (**D**,**K**) 0.17 µM DOX + 10 µM NITR, (**E**,**L**) 0.17 µM DOX + 25 µM NITR, (**F**,**M**) 0.17 µM DOX + 50 µM NITR, or (**G**,**N**) 0.17 µM DOX + 100 µM NITR. Scale bar: 50 µm; 100× total magnification.

Figure 9 shows the microscopic visualization of the MCF-7 breast cancer cell line and the SH-SY5Y cell line treated with the different combinations of DOX with NITR over a period of 48 h.

Through the results obtained for the combination of DOX with NITR (Figure 8 and Figure 9), we can observe that this combination of these two drugs was very effective for SH-SY5Y cells, since, for all tested combinations, a very low cell viability was reached (always below 40%) when compared to the individual drugs. For NITR, no decrease in cell viability was evident, which demonstrates that these two drugs together potentiated each other. For the MCF-7 cell line, slight decreases in cell viability were also observed, which shows that this combination was also beneficial for these cells; however, the increases in cell death observed were not as sharp as for the SH-SY5Y cells.

**Figure 10:**
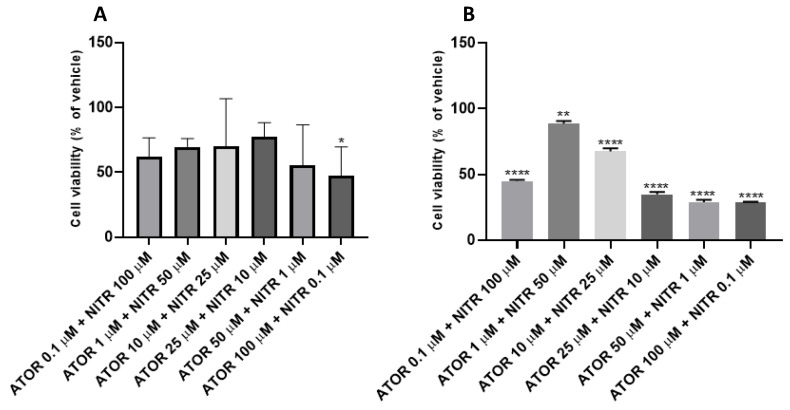
Effects of the combination of ATOR and NITR on cell viability of MCF-7 (**A**) and SH-SY5Y (**B**) cells. The cells were cultured in the presence of concentrations between 0.1 and 100 µM of ATOR and NITR. After 48 h, MTT assay was performed to measure cell viability. Values are expressed as percentages and represent means ± SEM. Each experiment was performed three times independently (n = 3). One-way ANOVA was used as statistical test. Statistically significant * *p* < 0.05, ** *p* < 0.01, and **** *p* < 0.0001 vs. vehicle.

To evaluate the different combinations of ATOR with NITR on the viability of MCF-7 and SH-SY5Y cells, cells were treated with ATOR and NITR at concentrations between 0.1 and 100 µM for 48 h. The percentage of cell viability was assessed by MTT assay (Figure 10).

**Figure 11:**
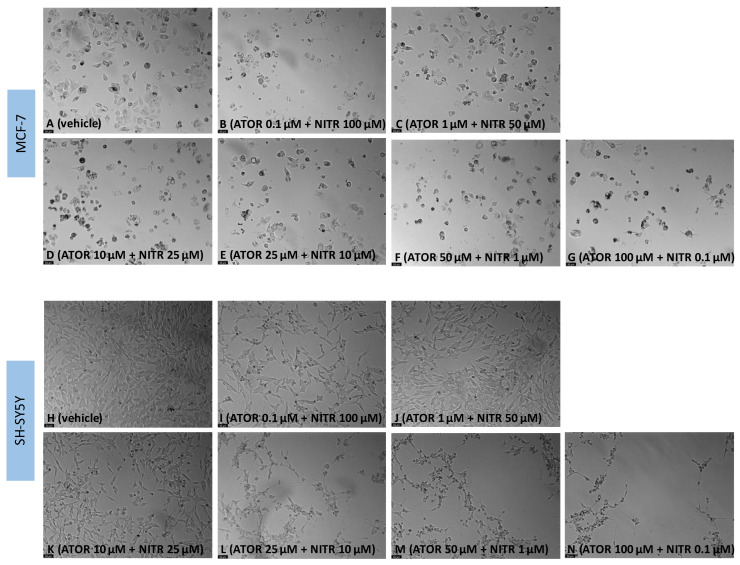
Microscopic visualization of the effects of combining ATOR with NITR on the morphology of MCF-7 cells for 48 h. Cells were treated with (**A**,**H**) 0.1% DMSO (control), (**B**,**I**) 0.1 µM ATOR + 100 µM NITR, (**C**,**J**) 1 µM ATOR + 50 µM NITR, (**D**,**K**) 10 µM ATOR + 25 µM NITR, (**E**,**L**) 25 µM ATOR + 10 µM NITR, (**F**,**M**) 50 µM ATOR + 1 µM NITR, or (**G**,**N**) 100 µM ATOR + 0.1 µM NITR. Scale bar: 50 µm; 100× total magnification.

Figure 11 shows the microscopic visualization of the MCF-7 breast cancer cell line and the SH-SY5Y cell line treated with the different combinations of ATOR with NITR over a period of 48 h.

Through the results obtained for the combination of ATOR with NITR (Figure 10 and Figure 11), it is visible that this combination was beneficial, since, for all combinations, there was a decrease in cell viability compared to the drugs separately. Observing Figure 10, it is possible to verify that, the combination of 0.1 µM ATOR with 100 µM NITR yielded the best effect. When compared with the individual results of these drugs (Figure 2 and Figure 4), we can affirm that, for this combination, there was a very sharp increase in cell death, since the cell viability of the drugs individually was around 108% for the concentration of 0.1 ATOR and 61% for the concentration of 100 NITR, whereas, when combined, these two drugs for these concentrations managed to achieve a cell death of about 38% for MCF-7 cells. For SH-SY5Y cells, the cell death of the individual drugs was around 7% for the concentration of 0.1 ATOR and 19% for the concentration of 100 NITR; when combined, these two drugs for this concentration achieved a cell death of about 55% for SH-SY5Y cells. Thus, we can state that both drugs potentiate each other; furthermore, for MCF-7 cells, NITR potentiates ATOR more than vice versa, whereas, for SH-SY5Y cells, it is ATOR that potentiates NITR. These results may be sustained by the effects of these drugs individually, demonstrated above.

**Figure 12:**
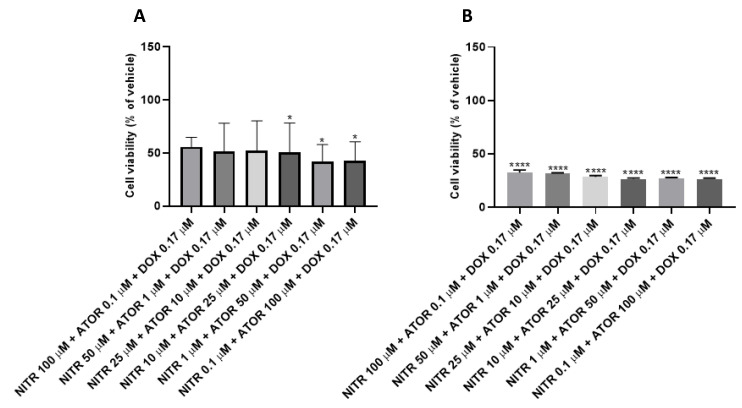
Effects of combining DOX with ATOR and with NITR on cell viability of MCF-7 (**A**) and SH-SY5Y (**B**) cells. The cells were cultured in the presence of concentrations between 0.1 and 100 µM of ATOR and NITR. After 48 h, MTT assay was performed to measure cell viability. Values are expressed as percentages and represent means ± SEM. Each experiment was performed three times independently (n = 3). One-way ANOVA was used as statistical test. Statistically significant * *p* < 0.05, and **** *p* < 0.0001 vs. vehicle.

The effects of different combinations of DOX with ATOR and with NITR were evaluated on the viability of MCF-7 and SH-SY5Y cells; for this purpose, cells were treated with 0.17 µM DOX and with concentrations between 0.1 and 100 µM ATOR and NITR for 48 h. The percentage cell viability was assessed by MTT (Figure 12).

**Figure 13:**
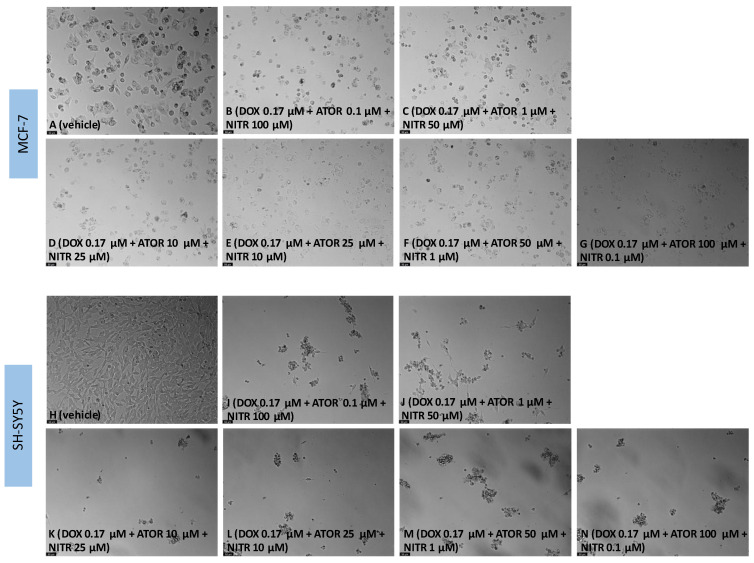
Microscopic visualization of the effects of combining DOX with ATOR and with NITR on the morphology of MCF-7 and SH-SY5Y cells for 48 h. Cells were treated with (**A**,**H**) 0.1% DMSO (control), (**B**,**I**) 0.17 µM DOX + 0.1 µM ATOR + 100 µM NITR, (**C**,**J**) 0.17 µM DOX + 1 µM ATOR + 50 µM NITR, (**D**,**K**) 0, 17 µM DOX + 10 µM ATOR + 25 µM NITR, (**E**,**L**) 0.17 µM DOX + 25 µM ATOR + 10 µM NITR, (**F**,**M**) 0.17 µM DOX + 50 µM ATOR + 1 µM NITR, or (**G**,**N**) 0.17 µM DOX + 100 µM ATOR + 0.1 µM NITR. Scale bar: 50 µm; 100× total magnification.

Figure 13 shows the microscopic visualization of the MCF-7 breast cancer cell line and the SH-SY5Y cell line treated with the different combinations of DOX with ATOR and with NITR over a period of 48 h.

Through the results obtained for the combination of DOX with ATOR and NITR (Figure 12 and Figure 13), we can observe that, for the three tested combinations, all managed to achieve lower cell viability compared to the cell viability of all drugs separately for both cell lines tested. From Figure 12, we can see that the combination that achieved the highest cell death for MCF-7 cells was 0.17 µM DOX with 50 µM ATOR and with 1 µM NITR, which reached a cell viability of about 42%; for SH-SY5Y cells, 0.17 µM DOX with 100 µM ATOR and with 0.1 µM NITR reached a cell viability of about 26%. Thus, we can observe that the combination of DOX with ATOR and NITR was able to further potentiate these drugs to achieve higher cell death, and we can conclude that the combination of DOX with ATOR and with NITR was quite good in reducing the viability of MCF-7 and SH-SY5Y cells; consequently, all drugs potentiated each other.

### 3.3. Synergistic Combinations of DOX and Repurposed Drugs

To investigate the effects of the combinations of DOX with the repurposed drugs, atorvastatin and nitrofurantoin, and of the repurposed drugs with each other, the combi-nation index (CI) was calculated according to the Chou–Talalay method using CompuSyn software. The Chou–Talalay method is based on the median effect equation, derived from the principle of the law of mass action. This unified theory encompasses the Michaelis–Menten, Hill, Henderson–Hasselbalch, and Scatchard equations in biochemistry and biophysics and provides a quantitative definition for additive effect (CI = 1), synergism (CI < 1), and antagonism (CI > 1) in drug combinations [25]. The fractional effect is a value between 0 and 1, where 0 means that the drug did not affect cell viability, and 1 means that the drug had a full effect in decreasing cell viability [19,26]. The combination of DOX with atorvastatin in MCF-7 cells did not show synergism for any of the combinations tested (Table 2), showing that these two drugs had an antagonistic action in these cells, with a CI greater than 1 for all pairs of combinations. For SH-SY5Y cells, this combination was very promising, since the combination of 0.17 μM DOX with 100 μM ATOR was the only one that did not show synergism, while all other synergistic pairs showed synergism in this cell line and an Fa value of 0.74 (Table 2).

For the combination of DOX with NITRO, for MCF-7 cells, this was the most promising combination for this cell line, with three synergistic pairs and an Fa value reaching 0.54 (Table 3); for SH-SY5Y cells, this combination was one of the most promising with all pairs of combinations being synergistic, i.e., with CI < 1 and with almost all Fa values reaching 0.65 (Table 3).

For the combination of ATOR with NITRO, in MCF-7 cells, this combination did not result in any synergism, with CI > 1 for all concentration pairs (Table 4); for SH-SY5Y cells, this combination resulted in four synergistic pairs, with an Fa value of 0.71 (Table 4).

Lastly, for the combination of DOX with ATOR and with NITRO, in MCF-7 cells, this combination did not show synergism in any of the combinations tested (Table 5); in SH-SY5Y cells, this combination was one of the most promising, with all synergistic pairs showing synergism, i.e., CI < 1 for all combinations tested (Table 5). These results, thus, demonstrated that NITRO and ATOR may be promising combinations.

**Figure 14:**
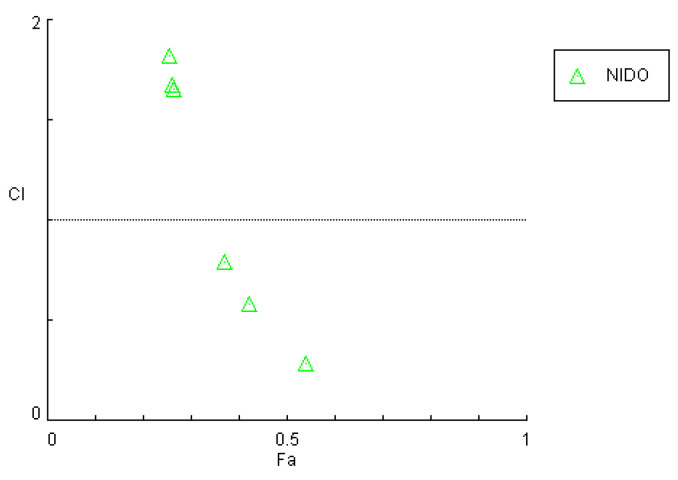
Fa–CI plot of combinations in MCF-7 cell line. Only the NIT + DOX combination (green) had CI values in this graph range of CI (0–2).

**Figure 15:**
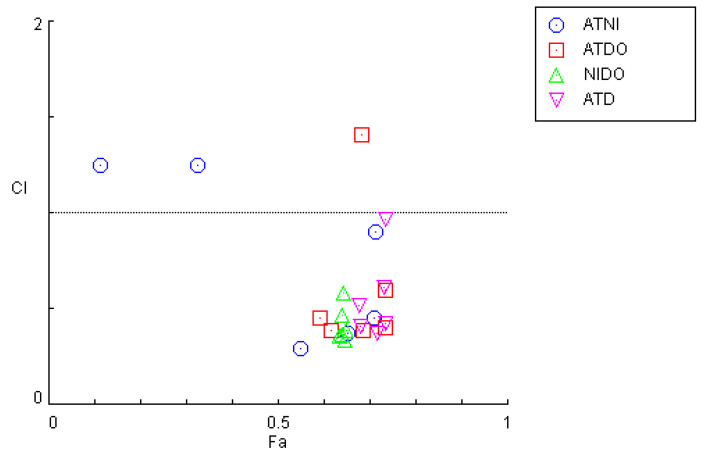
Fa–CI plot of combinations in SH-SY5Y cell line. Combinations: ATOR + NIT (blue), ATOR + DOX (red), NIT + DOX (green), and ATOR + NIT + DOX (pink).

Figure 14 and Figure 15 show the Fa–CI plots of the combinations in the MCF-7 and SH-SY5Y cell lines, respectively.

The dose reduction index (DRI) was also calculated; this index refers to the percentage of dose reduction for each drug within the combination that can be reduced to generate a specific effect as a result of the synergy. A DRI > 1 indicates a favorable dose reduction, while a DRI < 1 represents an unfavorable dose reduction, and a DRI = 1 shows no corresponding dose reduction. It is also necessary to mention that DRI is associated with CI, but it is only the CI values that effectively verify the synergism or antagonism of drug combinations. It should then be considered that, once the dose of a drug is reduced, the toxicity of this drug will eventually decrease [27].

**Figure 16:**
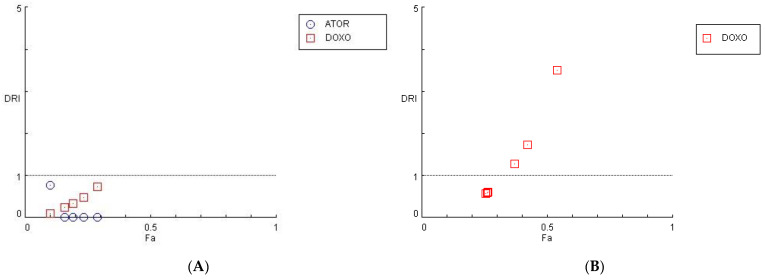

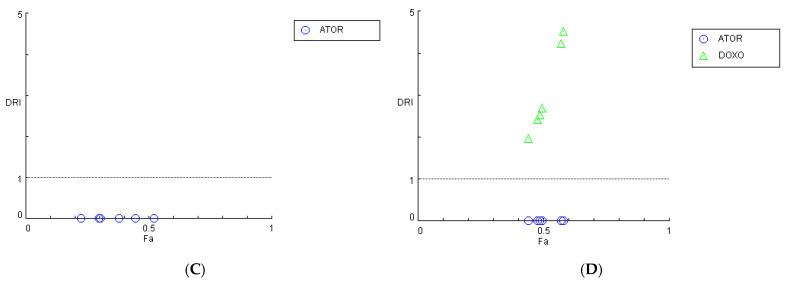
Fa–DRI plot of combinations in MCF-7 cell line. Combinations: ATOR + DOX (**A**), NIT + DOX (**B**), ATOR + NIT (**C**), and ATOR + NIT + DOX (**D**).

**Figure 17:**
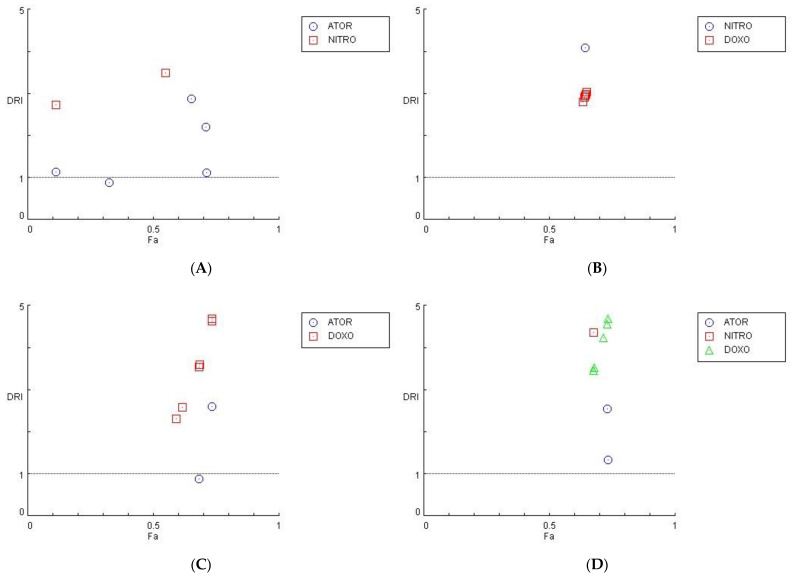
Fa–DRI plot of combinations in SH-SY5Y cell line. Combinations: ATOR + DOX (**A**), NIT + DOX (**B**), ATOR + NIT (**C**), and ATOR + NIT + DOX (**D**).

Figure 16 and Figure 17 show the Fa–DRI plots of the combinations in the MCF-7 and SH-SY5Y cell lines, respectively.

For MCF-7 cells, the combinations of DOX with ATOR and ATOR with NIT showed a DRI < 1 (Figure 16A,C), which indicates that there should be no dose reduction, i.e., these combinations show an unfavorable dose reduction. In contrast, the combinations of DOX with NIT and of DOX with ATOR and NIT (Figure 16B,D) had a DRI > 1, which shows that these combinations can benefit from favorable dose reduction.

For SH-SY5Y cells, for all combinations tested, a DRI > 1 was evidenced (Figure 17), which highlights that all these combinations in this cell line can benefit from a favorable dose reduction.

Through this synergy analysis, we demonstrated that the two repurposed drugs tested in this study can synergistically decrease cell viability when combined with DOX for SH-SY5Y cells. Our results revealed more synergistic pairs for SH-SY5Y compared to MCF-7 cells, with almost all the combinations tested resulting in synergistic pairs for the lowest concentrations. For MCF-7 cells, the results evidenced that almost all the tested combinations did not result in synergistic pairs; hence, ATOR and NITRO cannot synergistically decrease the cell viability of MCF-7 cells when combined with DOX.

Although the exact mechanism of its antiproliferative effects is currently unknown, atorvastatin both modifies the cell cycle and induces suppression of growth or apoptosis of malignant cells. Furthermore, the lipophilic nature of atorvastatin allows it to easily cross the cell membrane and induce these effects. Indeed, a previous study reported that ATOR treatment at concentrations of up to 80 μM caused a decrease in the viability of MCF-7 cells after 24 h and 48 h [12]. These results are in concordance with our results in which cell viability decreases were also observed for MCF-7 cells. The other repurposed drug, NTRO, is a synthetic antibiotic that has potential toxic effects attributable to the nitro group (NO_2_) attached to the furan ring. The nitro group gives this molecule a toxicophoric function, which acts as an electron acceptor, thus inhibiting enzymes involved in pyruvate metabolism, an essential pathway of cellular metabolism [11].

As explained above, SH-SY5Y cells are neuronal cells. These kinds of cells are known to be more sensitive to cytotoxic effects than breast cells. Indeed, this study is innovative because there are few reports about these drugs in these types of cells. Future studies focused on the molecular mechanisms underlying the differences between these cells regarding the obtained responses in this study are very important. Nevertheless, this study revealed the potential of drug combination and repurposing in the context of cancer treatment.

## 4. Conclusions

We concluded that ATOR had inhibitory effects on the viability of both tumor cell lines tested, MCF-7 and SH-SY5Y, and that NITRO showed inhibitory effects on the growth and viability of MCF-7 cells, while, in SH-SY5Y cells, this repurposed drug did not show any cytotoxic effects. Regarding the combination of DOX, the reference drug used in breast cancer, with the repurposed drugs, it is possible to conclude that, for all tested combinations, there was a reduction in cell viability and, consequently, an increase in cell death. Thus, DOX was able to potentiate ATOR and NITRO in both cells tested. Concerning the combination of ATOR with NITRO, it is possible to see that both drugs were able to potentiate each other, but that NITRO showed a greater potentiation on ATOR for MCF-7 cells; on the other hand, for human neuroblastoma cells (SH-SY5Y), the opposite occurred, i.e., ATOR showed a higher potentiation on NITRO, since it had no inhibitory effect on these cells when isolated and, when combined with ATOR, showed quite high cytotoxic effects. Through synergism, it was possible to conclude that the combinations of DOX with the repurposed drugs were more advantageous in SH-SY5Y cells than in MCF-7 cells, since, for all tested combinations, synergism was always evidenced for almost all studied combination pairs. This new drug combination model opens the door to a new pharmacological interaction between different reused drugs combined with each other or combined again but with a reference drug in oncology.

## Figures and Tables

**Table 1:** IC50 (half of the maximum inhibitory concentration) values for repurposed drugs atorvastatin and nitrofurantoin against MCF-7 and SY-SY5Y cells.

Drug	IC50 (MCF-7, µM)	IC50 (SH-SY5Y, µM)
Atorvastatin	37.98	10.10
Nitrofurantoin	5.70	>100

**Table 2:** Fractional effect (Fa) and combination index (CI) values ATOR and DOX combinations for 48 h in MCF-7 and SH-SY5Y cells. CI < 1 synergism, CI = 1 additivity, and CI > 1 antagonism. Fa values range from 0 (no cellular death) to 1 (complete cellular death).

Dose DOX (μM)	Dose ATOR (μM)	MCF-7		SH-SY5Y	
		Effect (Fa)	CI	Effect (Fa)	CI
0.17	0.1	0.15985	3.16E20	0.61633	0.38916
	1.0	0.19309	2.64E31	0.59243	0.45248
	10.0	0.23632	3.52E43	0.68567	0.38837
	25.0	0.10102	10.5377	0.73556	0.40282
	50.0	0.2349	8.02E43	0.73363	0.60022
	100.0	0.28865	1.88E56	0.68255	1.41110

**Table 3:** Fractional effect (Fa) and CI (combination index) values of NITRO and DOXO combinations for 48 h in MCF-7 and SH-SY5Y cells. CI < 1 synergism, CI = 1 additivity, and CI > 1 antagonism. Fa values range from 0 (no cellular death) to 1 (complete cellular death).

Dose DOXO (μM)	Dose NITRO (μM)	MCF-7		SH-SY5Y	
		Effect (Fa)	CI	Effect (Fa)	CI
0.17	0.1	0.26221	1.67662	0.63341	0.35821
	1.0	0.26396	1.65830	0.64722	0.33785
	10.0	0.25571	1.81996	0.64172	0.36880
	25.0	0.53983	0.28529	0.65123	0.38937
	50.0	0.37222	0.79532	0.64143	0.46750
	100.0	0.42196	0.58248	0.64410	0.58474

**Table 4:** Fractional effect (Fa) and CI (combination index) values of ATOR and NITRO combinations for 48 h in MCF-7 and SH-SY5Y cells. CI < 1 synergism, CI = 1 additivity, and CI > 1 antagonism. Fa values range from 0 (no cellular death) to 1 (complete cellular death).

Dose ATOR (μM)	Dose NITRO (μM)	MCF-7		SH-SY5Y	
		Effect (Fa)	CI	Effect (Fa)	CI
0.1	100.0	0.38063	1.71E71	0.55072	0.29008
1.0	50.0	0.30337	2.14E57	0.11475	1.24825
10.0	25.0	0.29898	2.68E57	0.32571	1.25105
25.0	10.0	0.22573	2.32E41	0.65215	0.37312
50.0	1.0	0.44593	4.03E85	0.71205	0.45641
100.0	0.1	0.52423	3.16E99	0.7135	0.8988

**Table 5:** Fractional effect (Fa) and CI (combination index) values of ATOR, NITRO, and DOX combinations for 48 h in MCF-7 and SH-SY5Y cells. CI < 1 synergism, CI = 1 additivity, and CI > 1 antagonism. Fa values range from 0 (no cellular death) to 1 (complete cellular death).

Dose ATOR (μM)	Dose NITRO (μM)	Dose DOX (μM)	MCF-7		SH-SY5Y	
			Effect (Fa)	CI	Effect (Fa)	CI
0.1	100.0	0.17	0.44234	1.89E82	0.67706	0.52120
1.0	50.0		0.48526	5.62E90	0.68137	0.40941
10.0	25.0		0.47763	2.68E90	0.71715	0.37664
25.0	10.0		0.49533	7.79E93	0.73526	0.42406
50.0	1.0		0.58165	1.8E109	0.73081	0.61436
100.0	0.1		0.57114	5.0E107	0.73571	0.96965

## Data Availability

Not applicable.
